# Human and Plant Fungal Pathogens: The Role of Secondary Metabolites

**DOI:** 10.1371/journal.ppat.1003859

**Published:** 2014-01-30

**Authors:** Daniel H. Scharf, Thorsten Heinekamp, Axel A. Brakhage

**Affiliations:** 1 Leibniz Institute for Natural Product Research and Infection Biology, HKI, Jena, Germany; 2 Friedrich Schiller University, Jena, Germany; Duke University Medical Center, United States of America

## What Are Secondary Metabolites?

Secondary metabolites (SM) are small organic molecules produced by various microorganisms (mainly fungi and actinobacteria, among others) through the action of large enzymes like nonribosomal peptide synthetases (NRPS) and polyketide synthases (PKS) or by enzymes like dimethylallyl transferases and prenyltransferases. These special metabolites are in general not essential for growth but are believed to be advantageous for the producing organism under certain conditions and in distinct habitats. In fungi, the genes responsible for SM biosynthesis are usually arranged in clusters [1], which are often found at the end of the chromosomes in subtelomeric regions [2], [3].

## Are Secondary Metabolites Essential for Fungal Pathogenicity?

**Figure 1:**
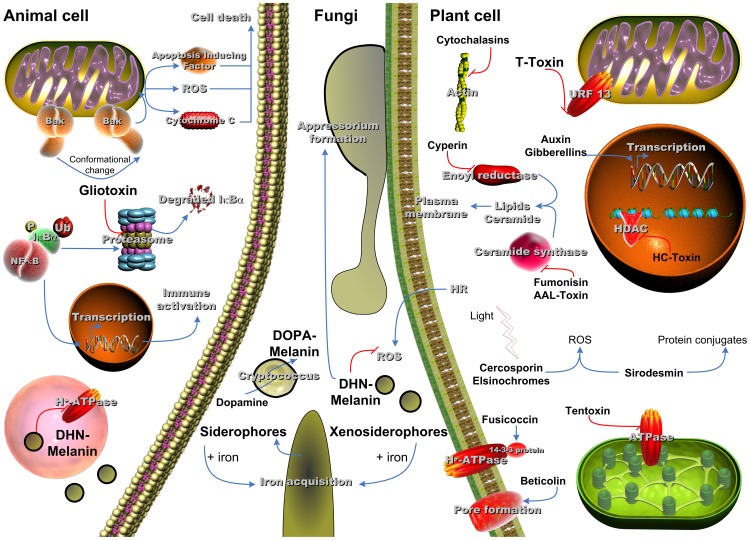
SM with influence on virulence of fungi in the respective host system. Animal cell: **Gliotoxin** acts on the mitochondrial protein Bak, which leads to apoptosis and inhibits the activation of NFκB through blocking proteasomal activity; **DHN-melanin** from *A. fumigatus* inhibits vH^+^-ATPase activity and therefore acidification of the phagolysosome, which counteracts killing of the conidia. Fungi: **Siderophores** are released by the fungus to ensure iron availability; **DHN-melanin** from plant pathogens is crucial for appressorium formation and counteracting reactive oxygen species (ROS) produced by the plant through hypersensitive response (HR) to prevent the spread of fungal infection; **DOPA-melanin** produced by *C. neoformans* from external precursors is an important part of the fungal capsule. Plant cell: **Cytochalasins** block cytokinesis; **T-Toxin** inhibits mitochondrial energy production; **Fumonisin**, **AAL-Toxin**, and **Cyperin** affect the membrane integrity; **Auxin** and **Gibberellins** act as phytohormones and alter transcription activity; **HC-Toxin** inhibits histone deacetylase; after light-driven activation both **Cercosporin** and **Elsinochromes** produce ROS, which damage the cell; **Sirodesmin** induces ROS and the formation of protein-conjugates; **Tentoxin** inhibits chloroplastidial energy production; **Fusicoccin** mediates irreversible stomata opening; **Beticolin** forms pores in the membrane and leads to leakage of the cell.

Yes, a few of these molecules have been shown to contribute to pathogenicity of several fungi (Figure 1). An excellent example of secondary metabolites that are essential for fungal pathogenicity is given by melanins [4]. What are melanins? Globally, they are defined as dark pigments of high molecular mass derived by oxidative polymerisation from phenolic precursors [5].

In fungi, two main types of melanin, dihydroxynaphthalene (DHN) melanin and dihydroxy phenylalanine (DOPA) melanin, and derivatives thereof have been reported to contribute to virulence. The ability to produce melanin via polymerisation of di- or tetrahydroxynaphthalene is widespread among fungi, and for some fungi the production of DOPA from L-tyrosine via dihydroxyphenylalanine has been shown. The function of melanins is extraordinarily wide. They exert beneficial functions to the producing organism as they mediate general protection against a wide variety of exogenous stresses. Melanins are often associated with the cell wall and thereby also contribute to fungal structures, such as ornamentation of the spore surface or pressure-stabilisation. Not surprisingly, melanins contribute to pathogenicity in several plant and human pathogenic fungi.

The two most prominent fungal organisms for which DHN-melanin is a prerequisite for virulence are the human pathogen *Aspergillus fumigatus* and the plant pathogen *Magnaporthe oryzae*. In *A. fumigatus*, DHN-melanin is mainly produced during conidiogenesis and accounts for the characteristic grey-green colour of the spores. These spores are ubiquitous in nature, with an estimation that every human inhales hundreds to thousands of conidia each day. This represents an important issue especially for patients suffering from an impaired immune system, making *A. fumigatus* the most important airborne fungal pathogen of humans [6].

In the lung, *A. fumigatus* conidia are confronted with the host's immune system from which phagocytes represent the first line of defense. Pigmentless white conidia are significantly reduced in virulence when tested in murine infection models. Fully pigmented conidia exert a dual effect on phagocytes: first, they prevent killing after phagocytosis by interfering with phagolysosomal acidification, and second, they inhibit apoptosis of macrophages. Thereby, *A. fumigatus* melanin is decisively pivotal in establishing a niche within phagocytes in which the fungus is protected from further immune reactions, providing the opportunity to grow out and hindering clearance of hyphae [3].

In the plant pathogenic fungus *M. oryzae* that causes the most important disease of rice worldwide, namely rice blast, the underlying mechanism by which DHN-melanin impacts virulence differs fundamentally from that of *A. fumigatus* [7]. Infection of rice plant leaves requires fungal penetration of the plant cell wall. The high turgor that is needed for this process is produced by a specialized DHN-melanin–containing fungal structure, the appressorium, from which the melanin layer hinders the efflux of solute [8]. Consequently, melanin-deficient *M. oryzae* mutants produce non-pigmented appressoria that are unable to penetrate epidermal cells of the leaf cuticle [9]. Targeting enzymes of the *M. oryzae* DHN-melanin pathway by chemicals such as carpropamid has been shown to efficiently prevent an infection.

*Cryptococcus neoformans*, the main opportunistic fungal infectious agent of patients suffering from AIDS, produces melanin exclusively via the L-dopa pathway from a variety of exogenously recruited precursers (e.g., L-DOPA, D-DOPA, dopamine, norepinephrine). Melanization occurs during infection, and mutants of *C. neoformans* lacking melanin pigment showed a reduced virulence in a murine infection model [4], [10].

Another good example of SM essential for fungal virulence is siderophores. As iron is an indispensable nutrient for all eukaryotic organisms, the ability to overcome iron limitation also represents a prerequisite for pathogenicity. Although iron is highly abundant in the oxidized form in the environment, the availability within a host, both animal and plant, is extremely low. For example, humans utilize carrier proteins like lactoferrin, transferrin, ferritin, and hemoglobin to bind iron, resulting in a final concentration of free iron of ∼10^−24^ M that is far from sufficient to meet a fungal pathogen's need to maintain its iron homeostasis during infection. As a countermove, fungal pathogens have evolved several strategies, amongst which is the production of siderophores for iron acquisition. Siderophores are small secreted molecules chelating iron with a complex formation constant that can reach >10^30^ M and thereby enable the fungal pathogen to capture iron from the host organism and even from stainless steel. Siderophores represent SM because their synthesis involves an NRPS, which also constitutes a promising drug target, as such enzymes are lacking in humans.

This struggle for iron obviously suggests a correlation of siderophore production and virulence of pathogenic fungi [11]. Again, *A. fumigatus* might serve as a good example of a human pathogenic fungus producing siderophores to cope with low iron availability in the host. For extracellular iron acquisition, *A. fumigatus* produces mainly the hydroxamate type siderophore triacetylfusarinine C (TAFC) by the NRPS SidD. Intracellular iron storage within *A. fumigatus* hyphae is mediated by the ferrichrome ferricrocin for which biosynthesis SidC is the core NRPS [12]. In conidia, hydroxyferricrocin is employed to guarantee iron supply during germination. Dissecting the pathways for intra- and extracellular siderophore biosynthesis pathways revealed their distinct cellular and disease-related roles during infection. For example, conidial hydroxyferricrocin is crucial, not only for developmental processes such as germ tube formation, sporulation, and stress resistance, but also for initiation of infection. By contrast, elimination of extracellular siderophores attenuates virulence but has no effect on germ tube formation.

With regard to animal–pathogen interaction, siderophore biosynthesis genes were found to be transcriptionally activated by confrontation with immune cells and during infection. Not surprisingly, *A. fumigatus* mutants in the siderophore biosynthesis gene *sidA*, which is essential for biosynthesis of a precursor for all secreted and intracellular siderophores, were even unable to initiate an infection in a murine model of pulmonary aspergillosis. Deletion of *sidC* or *sidD* caused at least partial attenuation of virulence [11].

The essential need to cope with low iron concentrations in the host is pertinent also for phytopathogenic fungi. Depending on the host-plant/pathogen combination, several secreted siderophores were identified as virulence determinants (*Fusarium graminearum*/rice, or *Alternaria brassicicola*/*Arabidopsis thaliana*). Notably, *M. oryzae* only produces an intracellular siderophore, ferricrocin, that also contributes to pathogenicity on rice by interfering with turgor generation by the appressorium essential for plant cell infection, as described above for melanin [13].

Besides producing their own iron chelators, some fungi, such as the pathogenic yeasts *Candida albicans* and *C. neoformans*, are also able to utilize xenosiderophores, iron-loaded siderophores originally produced by other microorganisms in the same habitat [14].

Taken together, melanins and siderophores are important pathogenicity factors in both plant and human pathogenic fungi because they are crucial for certain steps of pathogenesis like infiltration or survival.

## Can Secondary Metabolites Increase the Virulence of Fungal Pathogens?

There are several examples showing that SM contribute to the outcome of an infection but not to the onset of an infection. For some of these molecules, the mechanism of action is known (Figure 1). Such metabolites may naturally act as agents of competition or as communication signals in the environment and thus gain importance in certain pathogen–host interactions. Two SM produced by *Beauveria bassiana* provide excellent examples [15]. This fungus produces the SM bassinolide and beauvericin and is a facultative pathogen of insects with a broad host range. The abrogation of production of one of these molecules resulted in a decreased virulence in different insect infection models. The mechanism behind the action of bassinolide and beauvericin as virulence factors is unknown.

By contrast, the mechanism of host-selective T-toxin in the *Cochliobolus heterostrophus*/maize pathosystem is well established. T-toxin selectively affects mitochondria through the binding to the URF13 protein. This causes a conformational change in the polypeptide, leading to the formation of a pore in the mitochondrial membrane, which significantly damages the plant cells [16], [17].

Another example is gliotoxin (GT) produced by *A. fumigatus*, which was shown to attribute to virulence in mice immunosuppressed by exclusive administration of cortisone acetate [18], [19]. The GT cluster is up-regulated after confrontation with immune cells [2], [20]. The toxin can kill immune cells that normally clear the fungus from the lung epithelium. The toxicity of the molecule depends on its disulphide bridge, which is reduced after uptake in the host cell [21], [22]. Reduced GT can then inactivate essential proteins by the formation of protein-GT disulphides and initiate a redox cycle that permanently produces reactive oxygen intermediates, thereby harming the cell. Thus, the production of GT constitutes an advantage as soon as the fungus is confronted by immune cells or other enemies in the environment (Figure 1).

In conclusion, a few SM are essential for manifestation of an infection in animal or plant hosts. The two most important examples are melanins and siderophores, and the deletion of their key biosynthetic genes leads to attenuated and apathogenic strains, respectively. Other SM are, at least from the point of view of the pathogen, advantageous for the infection process and contribute in modulating the progress of a disease. Fungal SM act in different ways and increase the pathogen's ability to counteract adverse conditions in the host environment, irrespective of whether it is an animal or plant host. Since filamentous fungi encode between 30 to 70 secondary metabolism gene clusters (the products of most of these clusters are unknown) and there even exists cross-talk between clusters, resulting in the formation of hybrid molecules, it can be expected that up to 100 SM are produced by a single filamentous fungus. All of these compounds have the potential to contribute to pathogenicity. Thus, it will be very important to elucidate the nature and impact of these compounds on pathogenicity.
